# Investigating the Temporal Relationships between Symptoms and Nebuliser Adherence in People with Cystic Fibrosis: A Series of N-of-1 Observations

**DOI:** 10.3390/healthcare8010022

**Published:** 2020-01-21

**Authors:** Rosie Martin, Madelynne Arden, Jenny Porritt, Martin Wildman, Felix Naughton

**Affiliations:** 1 Centre for Behavioural Science and Applied Psychology, Sheffield Hallam University, Sheffield S10 2BQ, UK; m.arden@shu.ac.uk (M.A.); j.porritt@shu.ac.uk (J.P.); 2 Sheffield Teaching Hospitals, Sheffield S5 7AU, UK; martin.wildman@sth.nhs.uk; 3 School of Health Sciences, University of East Anglia, Norwich NR4 7TJ, UK; f.naughton@uea.ac.uk

**Keywords:** cystic fibrosis, adherence, N-of-1, ecological momentary assessment

## Abstract

Treatment adherence in adults with cystic fibrosis (CF) is poor. One of the reasons identified for lack of adherence to nebulised treatments is that patients may not experience any immediate relief in their symptoms or notice changes as a result of taking their treatment, thus many report that they do not perceive there to be consequences of non adherence. The aim of the study was to investigate the temporal relationships between symptoms and adherence to nebulised treatments in adults with CF using an N-of-1 observational design. Six participants were recruited for a six-week period during which time they completed a daily online respiratory symptom questionnaire. Adherence to treatment was measured throughout the duration of the study using an eTrack® nebuliser that logged date and time of treatments taken. Data generated from each participant was analysed separately. There were significant relationships between pain and adherence for three participants, tiredness and adherence for one participant and cough and adherence for one participant. For all of these findings, the symptom and adherence were experienced on the same day. Extending the monitoring period beyond six weeks may provide increased insight into the complex relationship between symptoms and adherence in CF.

## 1. Introduction

Cystic fibrosis (CF) is a life-limiting respiratory condition which affects over 10,000 people living in the UK [1], and over 44,000 patients in Europe [2]. The disease, which is caused by mutations in the transmembrane regulator (CFTR) gene, causes a build-up of thick and sticky mucus affecting different functions of the body with particular impacts on respiratory/pulmonary systems. Key respiratory symptoms include cough, sputum production, wheeze, chest tightness, difficulty breathing/shortness of breath and fever [3].

One of the respiratory complications associated with CF is exacerbations [4]. An exacerbation is indicated by a range of clinical signs and symptoms that indicate a need for additional [5] treatment usually with intravenous antibiotics [6]. Research findings have demonstrated an association between pulmonary exacerbations and declining lung function (measured as forced expiratory volume in one second: FEV1); FEV1 is one of the key outcomes for cystic fibrosis and other lung diseases [7]. Findings suggest that three exacerbations per year usually lead to a decline in a patient’s FEV1 [8].

Life expectancy of those living in the UK with CF is now around 47 years of age; this has increased in the last 30 years, when it was rare for a patient with CF in the UK to reach adulthood [9]. One of the reasons for this increase in life expectancy is due to development in effective medical treatments and therapies now available to people with CF [10]. One such example is nebuliser treatments; nebulisers convert medications into aerosol droplets that can be inhaled deep into the lungs [11].

Adherence to nebuliser treatments is poor [12] and there is a large difference between self-reported and objective adherence rates. Daniels et al. [13] found that adults self-reported 80% adherence levels, which corresponded with an adherence rate of only 36% for this group when using objective nebuliser reports taken from the I-neb adaptive aerosol delivery (AAD) nebuliser system (Philips Respironics; Chichester, England). The type of nebuliser treatment can also influence people’s treatment adherence. For CF patients given antibiotic nebuliser treatments, which are prescribed to treat chronic infections such as *Pseudomonas aeruginosa*, adherence has been reported to be between 31% and 53% [14]. Adherence to mucolytics, which are used to break down thick mucus, is reported to be higher, ranging between 53% and 79% [15].

Poor adherence to nebuliser treatments among CF patients has an adverse effect on their health, often causing a decline in lung function [16]. Poor adherence has also been linked to an increased number of hospital visits [17]. When adherence is high, this has been found to have lower healthcare costs for both inpatient treatment and prescribed medications [17,18]. In 2013, it was estimated that CF patients treated in the UK by the National Health Service (NHS) spent 103,453 days receiving intravenous antibiotics [12]. One factor which can influence this perception of necessity is experiencing nonreinforcement as a result of taking medication (e.g., no noticeable reduction in their symptoms); if patients cannot feel it working, then they are less likely to perceive it as necessary.

The Necessity–Concerns Framework [19] has been applied to long-term conditions in an attempt to explain why patients do not adhere to their treatment plan. It is theorised that people who adhere to medication have stronger perceptions regarding the necessity of the medication (e.g., health benefits) and fewer concerns relating to the adverse side effects associated with taking their prescribed treatments. This was highlighted by Sawicki, Heller, Demars and Robinson [20], who interviewed 20 pairs of CF youth patients (aged 16–21 years old) with their parents and found that patients thought their treatment “makes no difference” to how they felt, which affected their intentions to adhere. These findings have been supported by the work of Arden et al. [21], who found that some participants have “dysfunctional beliefs” in terms of believing that adhering to their nebuliser treatment has no impact upon their health. It was also found that some patients reported adhering to treatment when they experienced more symptoms [21], which could suggest a relationship between symptoms experienced and adherence to nebuliser treatments.

Surprisingly, there is a paucity of research investigating the relationship between adherence and symptoms in patients with CF. Drug trials and reviews have concluded that nebuliser treatments such as tobramycin (an antibiotic) and hypertonic saline (a mucolytic) often lead to an improvement in FEV1 (lung function) after an average time period of four weeks [22].

If patients with CF could be provided with feedback about how their adherence to treatment relates to the symptoms they experience, this could influence their perceptions regarding the importance (i.e., necessities) of the treatment and their subsequent adherence behaviours. This requires a method to measure the impact that daily symptoms have on treatment adherence. An observational N-of-1 design enables relationships between variables of interest to be identified for each individual with the analysis powered by the number of observations rather than the number of participants [23]. Ecological Momentary Assessment (EMA) is an approach to data collection that involves collating data relating to people’s behaviours and symptoms in real time, which allows the analysis of temporal relationships using an N-of-1 approach. This prospective method of data collection has the advantage of reducing memory bias (and thus increasing data accuracy) compared to when participants are asked to provide data retrospectively (e.g., asked to recall their health-related experiences over the past month, year, etc.) [24]. The use of daily diaries as a tool for monitoring and reporting day-to-day symptoms has therefore become increasingly popular. However, because prospective EMA methods, such as daily diaries, involve collecting data from participants on a frequent and repeated basis, it is important that this approach is acceptable to patients, given the existing burden of treatment in those with CF [25].

The current study aims to use novel methods such as daily electronic diaries and N-of-1 analysis to investigate the temporal relationships between symptoms and adherence in people with cystic fibrosis, with a particular focus on identifying the period of time needed to sufficiently monitor this relationship.

## 2. Materials and Methods

### 2.1. Design

The current study adopted an observational N-of-1 design, where data from each participant was analysed separately. The dependent variable was the participants’ adherence to nebuliser treatments, which was measured objectively via an eTrack® (Pari GmbH, Stanberg, Germany) nebuliser. The predictor variables were the symptoms of cystic fibrosis which were self-reported via a web-based questionnaire on a daily basis. A Patient Participant Involvement group (PPI group) was consulted and contributed to the design of the daily diary study.

### 2.2. Sample

In total, six participants were recruited. Recruitment within this population was difficult (Supplementary Materials S1), however, the sample was similar to previous work using N-of-1 methods [26].

All participants were recruited from Sheffield Teaching Hospitals (STH) and the following inclusion/exclusion criteria were adopted:

Inclusion criteria: CF patients aged 16 years and older, receiving treatment, using an eTrack® (Pari GmbH, Stanberg, Germany) nebuliser system, English-speaking and literate to ensure consent could be obtained. Participants were also required to own a smartphone and be part of the CFHealthHub Data Observatory.

Exclusion criteria: Those with CF who were under the age of 16, not using an eTrack® (Pari GmbH, Stanberg, Germany) nebuliser as a part of their daily treatment and not receiving treatment through STH. Patients who were in the late palliative phase of treatment (i.e., people in the end stage of their illness for whom the emphasis of care was comfort), pregnant or on the transplant list at the start of the study were also excluded from taking part in the study.

All patients consented were enrolled in the CFHealthHub data observatory. As part of this quality improvement initiative, all patients used an eTrack® (Pari GmbH, Stanberg, Germany) nebuliser, which records adherence in real time and uploads the data via Bluetooth onto the CFHealthHub web platform and stores it on a cloud. Patients in the CFHealthHub data observatory provide informed consent for their adherence data to be collected from specially adapted nebulisers and uploaded to a web platform where it can be shared with health professionals and used for other research projects.

Participants were purposively sampled from the following groups: good adherence (over 80% annual adherence) [27], poor adherence (under 80% annual adherence), where adherence is defined as the percentage of prescribed treatment taken, good lung function (FEV1 = >70%), poor lung function (FEV1 = <70%). This was to ensure the sample contained patients with both low and high adherence and poor and good lung function.

### 2.3. Ethics Statement

This study received ethical approval from London Bromley Research Ethics Committee (17/LO/1769) on the 16th November 2017, and the Health Research Authority (12th December 2017). Informed consent was taken from all participants who were involved in the study.

### 2.4. Daily Symptom Diaries

In total, seven symptoms were tracked using an online questionnaire accessed via a smartphone every day for a total of six weeks. This was split into two three-week blocks, with a midpoint break (ranging from 9 days to 38 days) to provide an opportunity for the research team to interview the participants about the acceptability of the approach (potential burden of research participation was explored) and confirm whether they would like to continue symptom tracking. Participants were asked to rate each symptom out of 10 (e.g.,0 = not at all and 10 = a great deal) using a visual analogue scale, at the same chosen time most appropriate for them each day (questions can be found in Table 1). The symptoms participants were asked to rate were cough, wheeze, difficulty breathing, pain, tiredness, mucus amount, mucus colour [3]. The questionnaire took a maximum of two minutes to complete on Qualtrics© (Qualtrics, Provo, Utah) (an online questionnaire software). Participants were sent a daily email containing the link to the questionnaire.

### 2.5. Adherence Data

The adherence data used in this study was recorded using the same method as previous work in the area [12]. Objective adherence to the eTrack® system (Pari GmbH, Stanberg, Germany) was recorded along with the patients’ prescription. This data was used to calculate a total number of doses and the proportion of prescribed doses taken per day (%). Therefore, it is possible to take over the prescribed dose (which would mean adherence is over 100%).

### 2.6. Analysis

Data was analysed using IMB’s SPSS version 24 (Armonk, NY: IBM Corp). Each participant’s data was analysed separately using an N-of-1 approach [26] where statistical analyses are powered by the number of observations rather than the number of participants. As there have not been N-of-1 studies previously undertaken in this patient group looking at correlates of adherence, and within-person associations are likely to vary between individuals, it is challenging to accurately estimate the required number of observations for a given power level. However, it is suggested that 50 observations should provide sufficient power [28].

A missing value analysis was conducted on all symptom questions for all participants. Little’s Missing Completely at Random test revealed all data was missing completely at random and therefore no cause for concern. Multiple imputation was performed in SPSS to deal with any missing values, as used by others undertaking similar N-of-1 analyses [26,29]. There was no missing data for the objective measurement of adherence.

As there was a break for all participants after a three-week period, two additional data points of missing data were included for all participants; this was to ensure data points were not linked artificially. Adherence was measured during this period, but symptom scores were missing as no data was inputted on these days.

Descriptive statistics were calculated for each symptom and adherence. Sequence charts were produced for each symptom (cough, wheeze, difficulty breathing, pain, tiredness and mucus amount) and also for objective adherence scores for the six-week symptom-tracking period to show the temporal relationship between variables.

A “prewhitening” procedure was applied to each data set as required; this was to ensure that autocorrelation was removed between data points [26].

To investigate relationships between symptoms and adherence, cross-correlations were examined in line with the recommendations of Naughton and Johnston [30]. If there was evidence of a relationship on cross-correlation charts (above the 95% confidence interval), a linear regression analysis was conducted to determine statistical significance (Supplementary Materials S2). The analysis was exploratory in nature to explore the cross-correlations identified rather than testing specific associations which were determined a priori.

## 3. Results

Six adults were recruited; baseline data are in Table 2.

### 3.1. Observation Points and Compliance

The majority of participants had over a 70% daily completion rate, and the number of observation points per participants ranged from 188 to 308. Two participants completed the self-monitoring diary every day; the largest amount of missing data was 38%. The mean daily completion rate for all participants was *M* = 87.6% (*SD* = 15.52). Daily completion rates for all participants can be seen in Table 3 below.

### 3.2. Descriptives Statistics and Autocorrelation

In total there were over 1,500 data series points; all symptom variables for all participants were prewhitened by one day to control for autocorrelation or in some cases as a precautionary measure in case of undetected autocorrelation. Descriptive statistics are presented in Table 4 below.

### 3.3. The Relationship between Symptoms of Cystic Fibrosis and Adherence to Nebulised Treatment

A summary of the results is presented in Table 5.

#### 3.3.1. Cough

Cross-correlation charts for participants 1, 3, 4 and 6 were not suggestive of a relationship; therefore, no further analysis was undertaken.

For participant 2, cross-correlation charts were indicative of a relationship between adherence and cough on day zero (*rlag0 = −.338).* To investigate this further, a linear regression was conducted which revealed a statistically significant negative relationship between coughing and objective nebuliser adherence on the same day (*F = 5.30; B = −8.95; 95% CI –16.80– –1.10; R^2^ = 0.115; P = 0.026).* This suggests that higher coughing and lower adherence occur on the same day for this person.

For participant 5, cross-correlation charts were indicative of a positive correlation at lag -2 (*rlag-2 = 0.336).* However, when the relationship was tested using a linear regression, it was not statistically significant (*F = 0.407; B = 5.70; 95% CI –12.33–23.74; R^2^ = −0.014; P = 0.527).*

#### 3.3.2. Wheeze

Cross-correlation charts for participants 1, 2, 3 and 4 were not suggestive of a relationship; therefore, no further analysis was undertaken. Participant 6 reported wheeze as being a zero for the duration of the study; therefore, no further analysis was undertaken.

For participant 5, there was indication of a relationship between the variables on the cross-correlation chart, *rlag-5 = −0.392*; a linear regression was conducted to determine the relationship between wheezing and adherence for participant 5, which was found to be nonsignificant (*F = 0.997; B = −9.80, 95% CI −29.65–10.05; R^2^ = 0.025; P = 0.324.*

#### 3.3.3. Difficulty Breathing

Cross-correlation charts for participant 2,3,5 and 6 were not indicative of any relationship between the variables difficulty breathing and adherence; therefore, no further analysis was undertaken. Participant 4 reported difficulty breathing as being at zero for the duration of the study; therefore, no further analysis was undertaken. Therefore, there was only one participant (participant 1) who presented with evidence of a relationship between difficulty breathing and nebuliser adherence.

For participant 1, cross-correlation charts revealed evidence of a relationship between difficulty breathing and adherence at lag 6 (*RLag6 = 0.446*). A linear regression revealed that this relationship was not statistically significant (*F = 0.059; B = 2.70; 95% CI −19.95–25.36; R^2^ = 0.002; P = 0.810)*. These findings would suggest that for all participants in the study, there was no significant relationship between experiencing difficulty breathing as a symptom and adherence to nebuliser treatments.

#### 3.3.4. Pain

Participant 6 reported pain as being a zero for the duration of the study; therefore, no further analysis was undertaken. Cross-correlation charts revealed no evidence of a relationship for participant 1 and 3; therefore, no further analysis was undertaken.

Cross-correlation charts for participant 2 revealed a moderate negative correlation between adherence and pain (*RLag0 = −0.311).* This relationship was found to be statistically significant when analysed using a linear regression (*F= 4.40; B = -4.46; 95% CI −8.76– –0.164; R^2^ = 0.97; P = 0.042)*. This suggests that participant 2 experiences higher pain and lower nebuliser treatment adherence on the same day.

Participant 4′s cross-correlation charts revealed a significant relationship between pain and adherence, *Rlag-2 = 0.36* and *Rlag0 = 0.34.* The variables (lag = 0) were analysed using a linear regression which revealed a significant relationship (*F = 4.40; B = 15.11; 95% CI 0.553–29.69; R^2^ = 0.102; P = 0.042)*. However, when the lag at −2 was investigated further, the regression revealed a nonsignificant relationship (*F = 2.13; B = 13.70; 95% CI –5.33–32.73; R^2^ = 0.056; P = 0.153*). This suggests only the relationship on the same day was significant; therefore, higher pain was associated with lower adherence.

For participant 5, cross-correlation charts revealed evidence of a relationship between pain and adherence for this participant *(RLag0 = −0.467; RLag-5 = −0.438).* A linear regression revealed that this relationship (lag = 0) was statistically significant (*F = 12.00; B = −43.81; 95% CI –69.32– –18.30; R^2^ = 0.218; P = <.001)*. However, when the lag at –5 was investigated further, this relationship was not significant (*F = 0.044; B = 3.12; 95% CI –26.90–33.15.1; R^2^ = –0.024; P = 0.834)*. These findings suggest there is a significant moderate relationship between adherence and pain on the same day for this participant, suggesting when adherence is lower, pain is higher.

#### 3.3.5. Tiredness

Cross-correlation charts revealed no evidence of a relationship between tiredness and adherence for participants 1, 3, 2, 4 and 6; therefore, no further analysis was undertaken.

Participant 5 cross-correlation charts for tiredness and adherence were indicative of a possible negative relationship at Lag 0 *(rlag0 = −0.319).* A linear regression revealed a significant relationship between tiredness and adherence (*F = 4.87; B = −7.87; 95% CI –15.00– -0.682; R^2^ = 0.102; P = 0.033)*. This suggests that for participant 5, there is an association between higher levels of tiredness and lower adherence to nebuliser treatment on the same day.

#### 3.3.6. Mucus Amount

Cross-correlation charts for participants 1, 4, 5 and 6 revealed no evidence of a relationship between mucus amount and adherence; therefore, no further analysis was undertaken.

Cross-correlation charts for participant 2 were suggestive of a medium negative correlation at 2-day lag (*rlag-2 = –0.470),* which would suggest mucus predicts adherence in 2 days’ time. However, when this was investigated further using a linear regression, this relationship was not statistically significant (*F = 0.735; B = 5.71; 95% CI –7.76–19.18; R^2^ = 0.018; P = 0.396)*.

Cross-correlation charts for participant 3 were suggestive of a correlation at –1 and 5-day lag (*rlag-1 = –0.350; rlag5 = −0.343).* However, when this was investigated further using a linear regression, this relationship was not statistically significant for lag −1 (*F = 1.14; B = −2.87; 95% CI –8.28–2.54; R^2^ = 0.027; P = 0.291)* or lag 5 (*F= 0.778; B = −2.84; 95% CI –9.37–3.69; R^2^ = 0.022; P = 0.384)*.

## 4. Discussion

The current study aimed to investigate the relationship between symptoms and adherence over a six-week period, in people with cystic fibrosis. The study offers mixed findings; for three participants, there was no evidence of a relationship between symptoms and adherence. For the remaining three participants, there was evidence that higher levels of cough, pain and tiredness were related to lower levels of adherence on the same day.

The lack of relationship between symptoms and adherence over a six-week period for some participants is surprising. Symptom reports are often used in drug trials to assess the impact of new treatments [31]. Perhaps these reports, in which symptoms are reported retrospectively rather than daily, overestimate the changes experienced, as has been found previously [31]. In addition, it is not clear how much adherence is necessary and for how long to produce symptom changes, and it is likely that there will be individual differences in the response. For same-day relationships, it is difficult to establish whether symptoms preceded adherence or adherence to treatment affected symptom experience that same day. Some patients report that treatment leads to a noticeable improvement in symptoms as a result of adherence [32]. Others report that they perceive less need for treatment when they are feeling well [33].

Due to the exploratory nature of the study and the investigation of cross-correlations, there is a risk of identifying some spurious associations between variables.

Previous research findings in the area have suggested one reason for poor adherence in this population could be that some patients do not notice the impact medication has on their symptoms and therefore feel it is not needed to treat their condition [19]. As theorised by Horne and Weinman [19], if a health behaviour is not viewed as a necessity, as humans we are much less likely to engage in the behaviour. In a meta-analysis of various long-term health conditions, Horne et al. [33] confirmed this link between higher medication adherence and a strong perception of the necessity of prescribed treatments.

If patients can be provided with feedback about how their treatment has improved their symptoms over time, this may help encourage these individuals to better adhere to treatment programmes. However, the results of this study indicate that the relationship between self-reported symptoms and adherence is not always clear. CF charities in both the UK and the USA are keen to explore the potential of digital devices to generate data that can provide insights into self-care that have the potential to promote good patient–clinician communication and create the opportunity to discuss experiences and challenges. Recent work in the UK has demonstrated that patients do engage well with such apparatus [18]. The aspiration to establish and develop such programmes emphasise the great interest in applications of using digital healthcare technology to help manage adherence to treatments in those with CF which is an issue high on the agenda with CF care.

Although it is evident that digital technology is becoming more popular in healthcare, the results of the current study suggest that a six-week monitoring period is not a sufficient amount of time to notice fluctuation in symptoms in all individuals. Therefore, a longer monitoring period would enable further investigation of the complex relationship between adherence behaviour and symptoms. Perhaps most importantly, what is not yet clear is if there will be relationships between symptoms and adherence that are easy to see and therefore convincing for patients. Without this understanding, this type of monitoring is unlikely to promote adherence.

One strength of the current study was that nebuliser adherence data was objective and taken directly from a chipped nebuliser system. It has been argued that such methods are more reliable than collecting adherence subjectively [34], which has associated drawbacks such as social desirability bias and retrospective bias. However, the study is not without limitations; for example, as discussed in the method, there was a break between the two symptom-tracking periods. Though this is not perhaps ideal, the reason for the break was to assess the acceptability of self-monitoring and potential burden of the study and was considered a high priority. Additionally, although daily diaries can identify temporal relationships between variables, in the current study, the relationship between adherence and symptoms typically was strongest on the same day. Alternative methods such as qualitative interviews could be adopted to help us investigate the nature of the relationship between symptoms and adherence further.

To conclude, the current study provides insight into the temporal relationships between symptoms and adherence in people with CF. These findings demonstrate that for three of six participants, symptoms such as cough, pain and tiredness were associated with adherence. The study provides evidence that a longer period of symptom tracking is required. The study utilises reliable objective adherence data which also demonstrates how novel methods such as N-of-1 can be used successfully for health-related self-monitoring in people with CF.

## Figures and Tables

**Table 1:** Questions presented to participants.

The following questions were presented to participants who were asked to respond on a 0–10 visual analogue scale:
Have you been coughing today?
Have you been wheezing today?
Have you experienced difficulty breathing today?
How is your pain today?
How is your tiredness today?
How much mucus have you had to cough up today?
The final question was asked as a Likert Scale:
What colour has your mucus been most of today? Clear, yellow/green, green with blood, other.

**Table 2:** Demographics and nebuliser treatments of participants.

Participant Number	Sex	Predicted lung function (%)	Baseline adherence (%)	Nebuliser treatment	Total Doses of Treatment Prescribed per Day
1	Female	89%	73%	2 mucolytics	3
2	Female	89%	99%	2 mucolytics	3
3	Female	73%	15%	1 mucolytic and 1 antibiotic	5
4	Male	80%	68%	2 mucolytics and 1 antibiotic	9
5	Female	96%	100%	1 mucolytic and 1 antibiotic	6
6	Female	45%	41%	1 mucolytic and 1 antibiotic	6

**Table 3:** The number of days each participant tracked symptoms for (%).

**Daily completion rate (%)**	**P1**	**P2**	**P3**	**P4**	**P5**	**P6**
	71%	62%	88%	100%	88%	100%

**Table 4:** Means (standard deviations) for symptom scores and adherence.

Participant Number	Cough	Wheeze	Difficulty breathing	Pain	Tiredness	Mucus amount	Objective adherence (%)	Range of Weekly Adherence*
*P1*	*3.85 (0.99)*	*0.10 (0.34)*	*1.22 (0.93)*	*0.038 (0.15)*	*3.47 (0.96)*	*2.93 (0.94)*	*82.5 (52.19)*	*71.5–100%*
*P2*	*0.26* *(0.40)*	*0.19* *(0.30)*	*0.48* *(0.54)*	*1.52* *(1.03)*	*4.26* *(1.57)*	*0.15* *(0.28)*	*97.55* *(10.26)*	*94.3–100%*
*P3*	*5.49* *(2.42)*	*2.80* *(1.88)*	*4.02* *(1.64)*	*2.93* *(1.07)*	*6.28* *(1.86)*	*4.23* *(2.22)*	*16.67 (35.66)*	*0-47.6%*
*P4*	*1.47 (0.83)*	*0.09 (0.30)*	*0.00 (0.00)*	*0.53 (0.70)*	*1.41 (1.11)*	*1.44 (0.80)*	*75.30* *(29.85)*	*52.3–100%*
*P5*	*2.55 (0.53)*	*0.25 (0.48)*	*0.22 (0.47)*	*0.08 (0.32)*	*1.94 (1.31)*	*2.28 (0.57)*	*87.78* *(27.80)*	*71.4–100%*
*P6*	*1.11 (1.22)*	*0.00 (0.00)*	*0.39 (1.13)*	*0.00 (0.00)*	*0.32 (0.86)*	*0.73 (0.66)*	*45.64 (36.04)*	*0–78.6%*

* For the duration of the study. All symptom scores are rated out of ten.

**Table 5:** Relationship between symptoms and adherence for all participants (regression analysis).

Participant	Cough and adherence	Wheeze and adherence	Difficulty breathing and adherence	Pain and adherence	Tiredness and adherence	Mucus and adherence
1	No relationship	No relationship	*Lag 6 = 0.446* *p = 0.810*	No relationship	No relationship	No relationship
2	*Lag 0 = −0.338 *p = 0.026*	No relationship	No relationship	*Lag 0 = −0.311 *p = 0.042*	No relationship	*Lag -2 = −470* *p = 0.396*
3	No relationship	No relationship	No relationship	No relationship	No relationship	*Lag 5 = −0.350* *p = 384* *Lag -1 = −0.343* *p = 0.291*
4	No relationship	No relationship	-	*Lag 0 = 0.339 *p = 0.025* *Lag -2 = 0.362* *p = 0.153*	No relationship	No relationship
5	*Lag -2 = 0.336* *p = 0.527*	*Lag -5 = −0.392* *p = 0.325*	No relationship	*Lag 0 = −0.467 *p = <.001* *Lag -5 = −0.438* *p = 0.834*	*Lag 0 = −0.319 *p = 0.033*	No relationship
6	No relationship	-	No relationship	-	No relationship	No relationship

Key: *Significant at *P* = 0.05, no relationship = did not pass 95% confidence interval, - = rated as constant zero.

## Supplementary Materials

The following are available online at https://www.mdpi.com/2227-9032/8/1/22/s1, Figure S1: CONSORT diagram; Figure S2: Cross-correlation charts
