# Supplementary material for: Investigating the Temporal Relationships between Symptoms and Nebuliser Adherence in People with Cystic Fibrosis: A Series of N-of-1 Observations
Source: Healthcare (Basel). 2020 Jan 21;8(1):22. doi: 10.3390/healthcare8010022 (PMC7151352; doi:10.3390/healthcare8010022)
Supplement: Supplementary file 1 [file healthcare-08-00022-s001.pdf]

Cross-correlations

Participant 1

Difficulty breathing

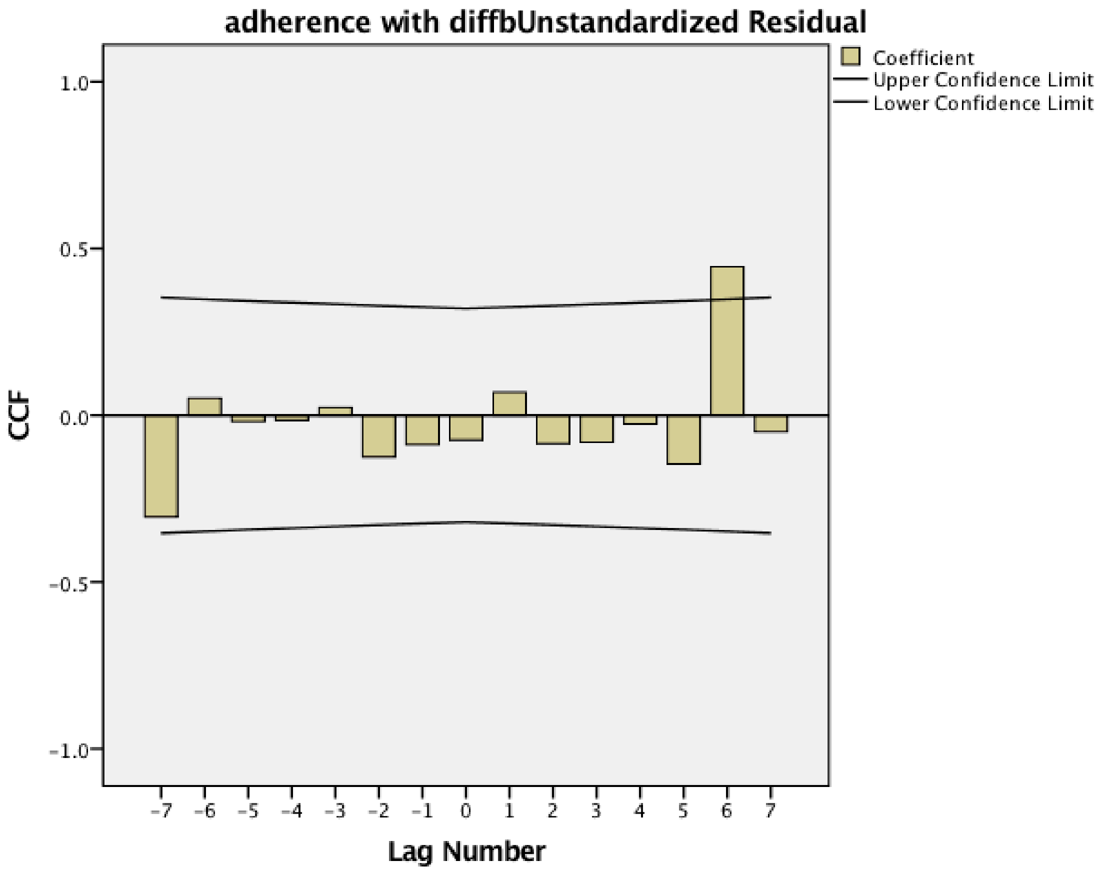

Participant 2

Cough

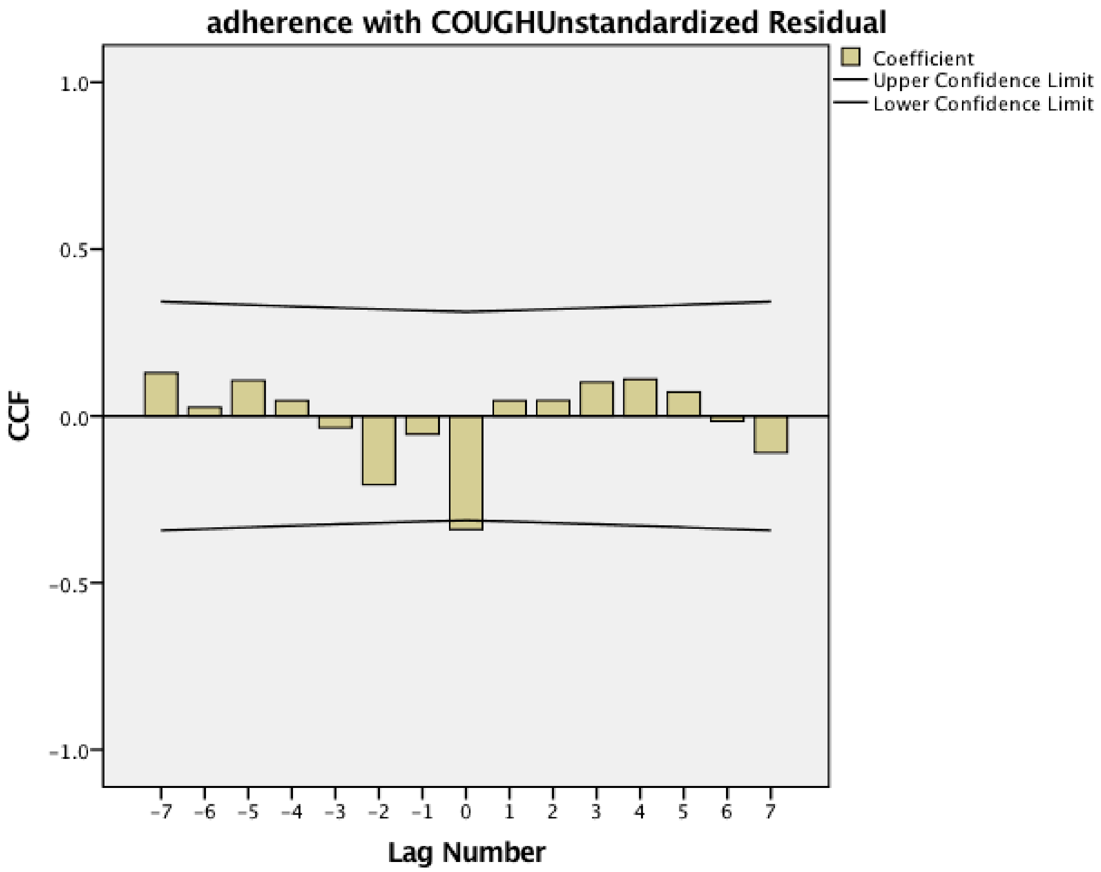
adherence with COUGHUnstandardized Residual
Coefficient
Upper Confidence Limit
Lower Confidence Limit
CCF
1.0
0.5
0.0
-0.5
-1.0
-7
-6
-5
-4
-3
-2
-1
0
1
2
3
4
5
6
7
Lag Number

Pain

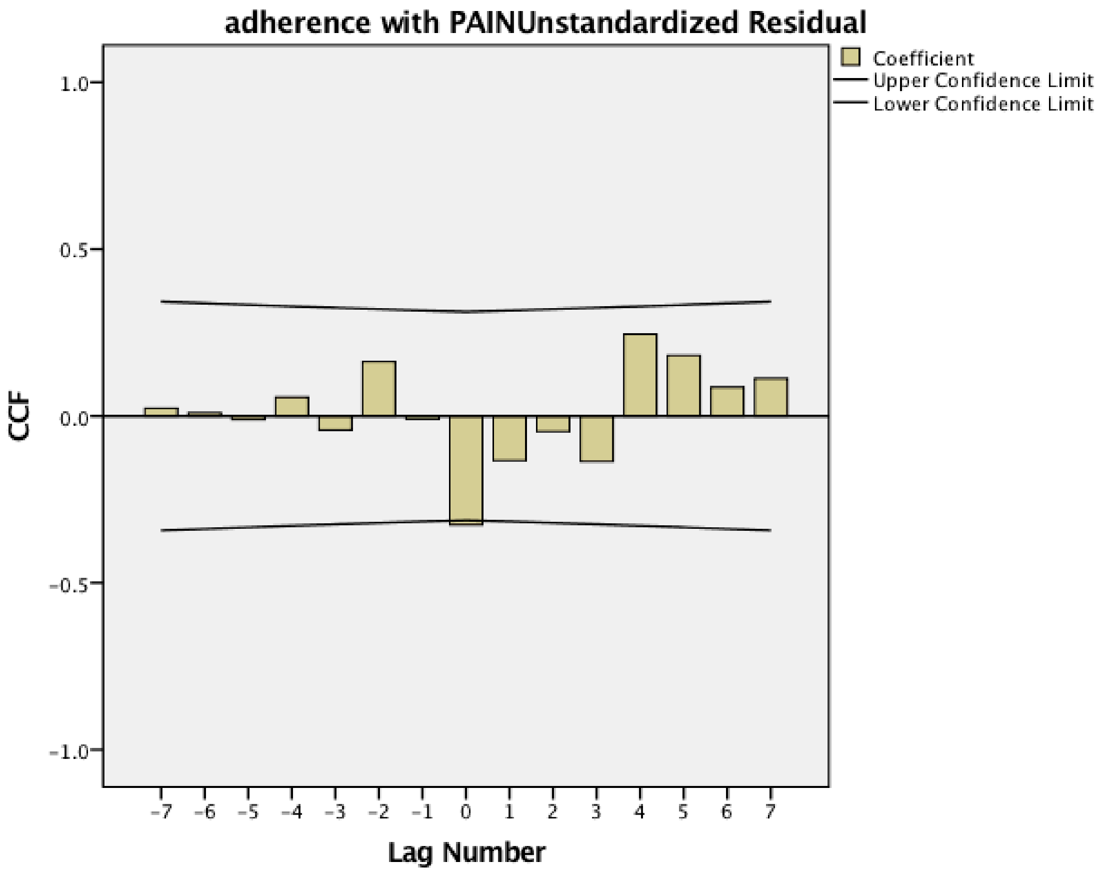
adherence with PAINUnstandardized Residual
Coefficient
Upper Confidence Limit
Lower Confidence Limit
CCF
1.0
0.5
0.0
-0.5
-1.0
-7
-6
-5
-4
-3
-2
-1
0
1
2
3
4
5
6
7
Lag Number

Mucus

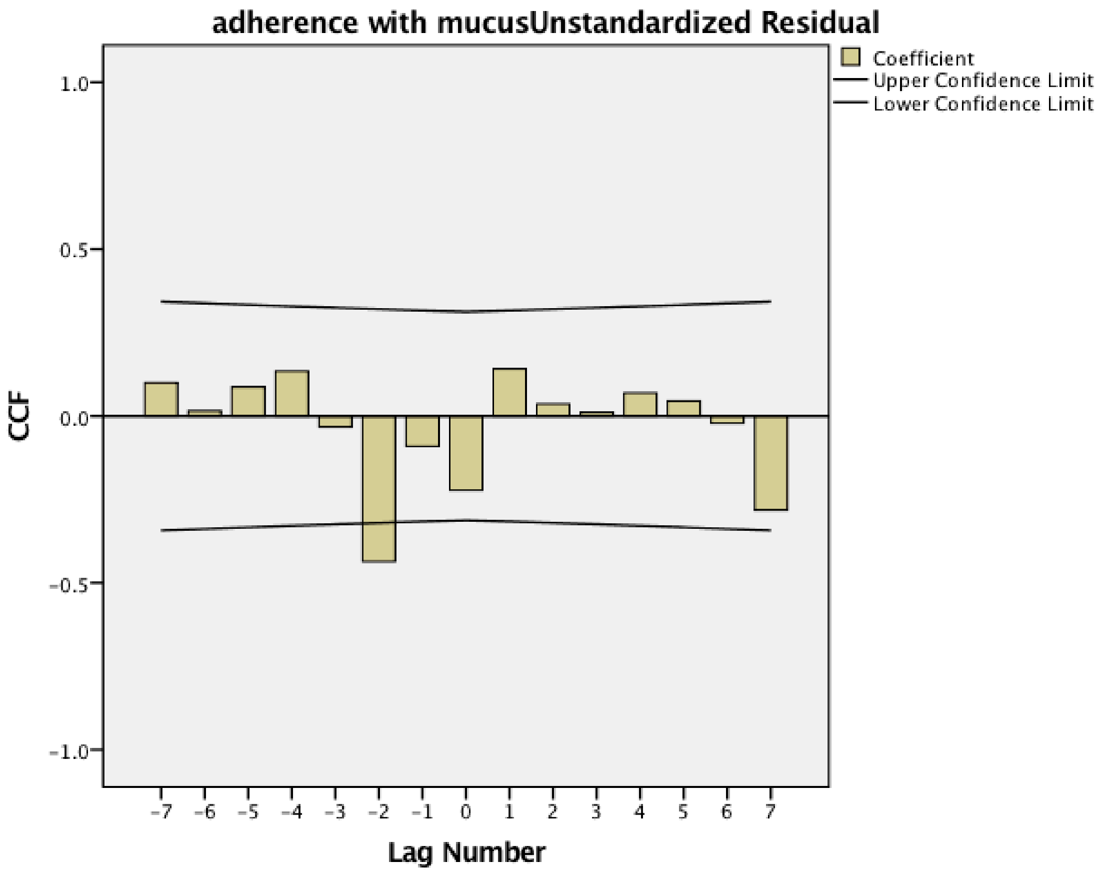

Participant 3

Mucus

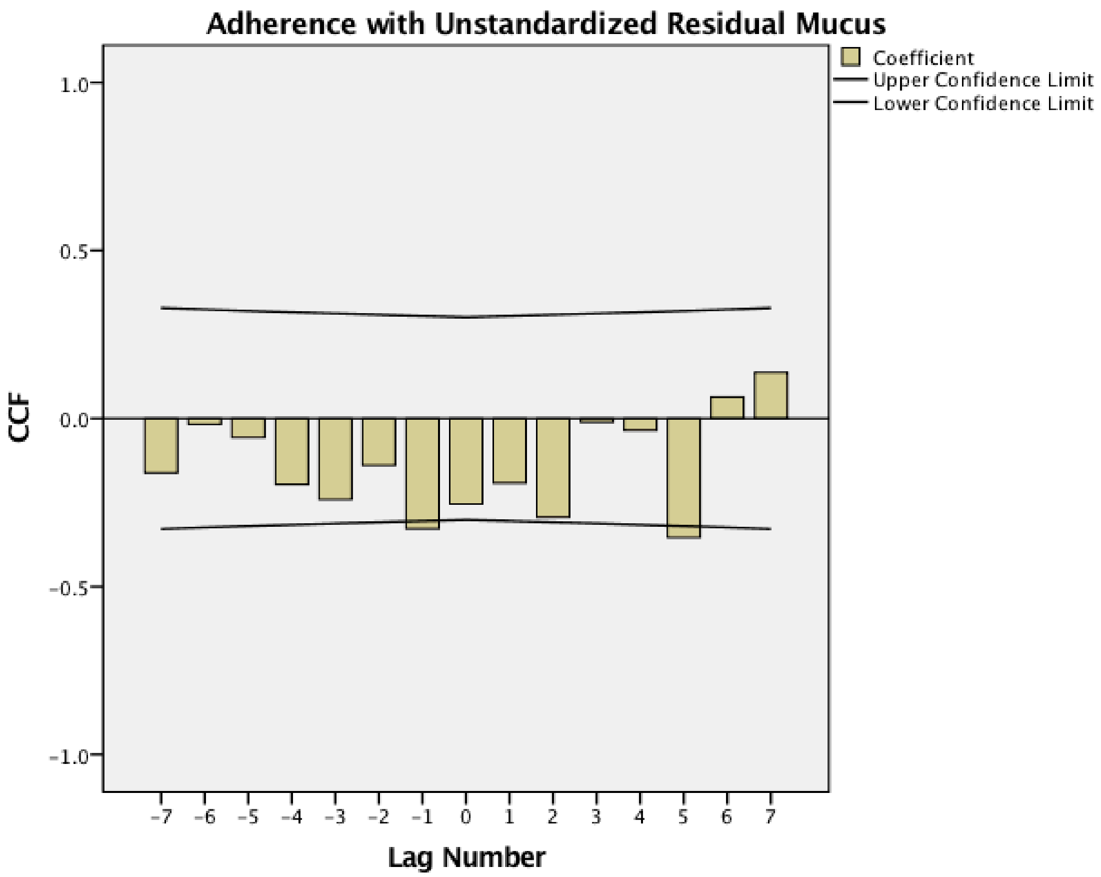

Participant 4

Pain

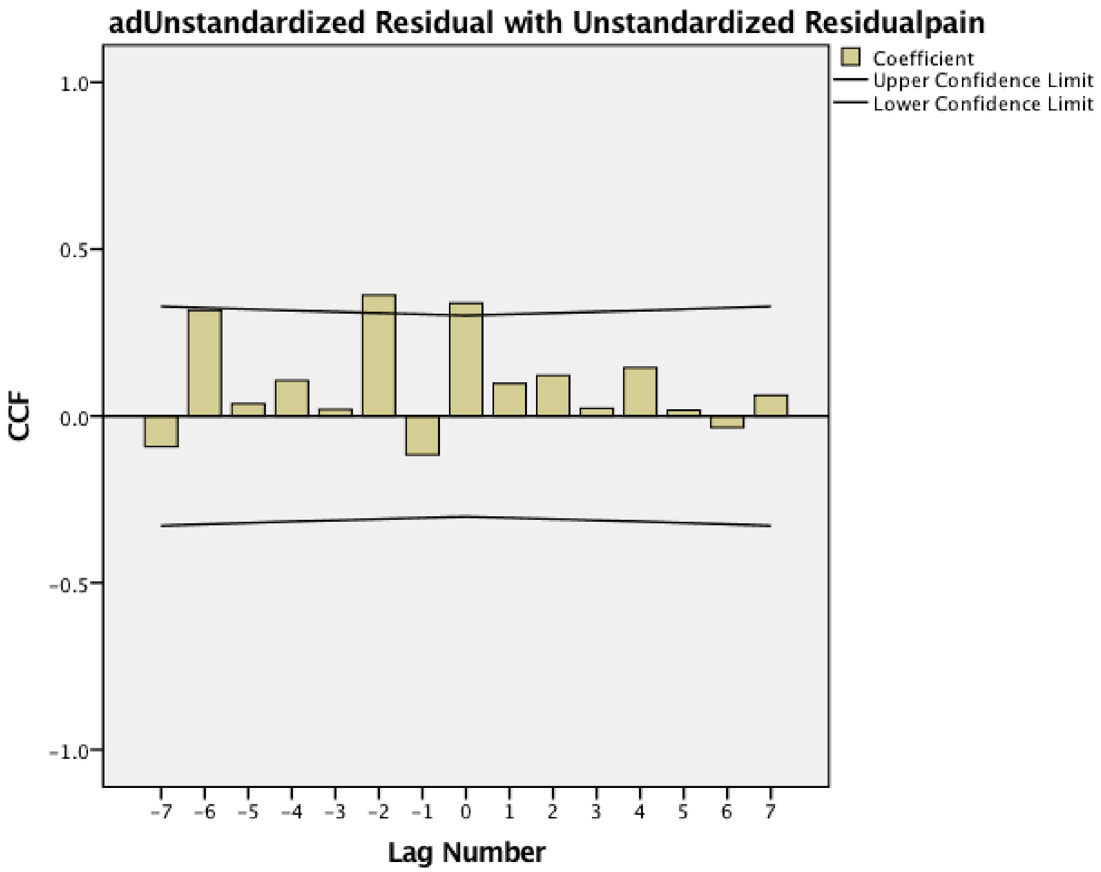

Participant 5

Cough

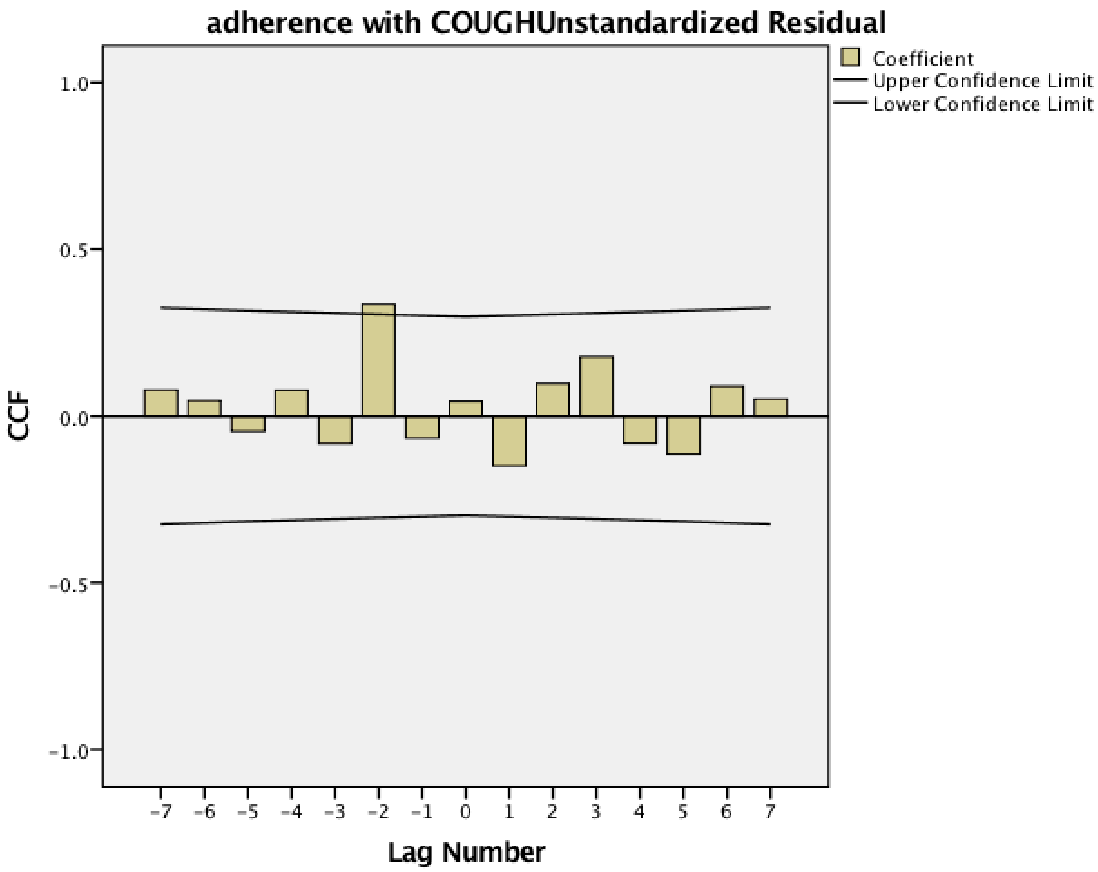
adherence with COUGHUnstandardized Residual
Coefficient
Upper Confidence Limit
Lower Confidence Limit
1.0
0.5
0.0
-0.5
-1.0
CCF
-7
-6
-5
-4
-3
-2
-1
0
1
2
3
4
5
6
7
Lag Number

Wheeze

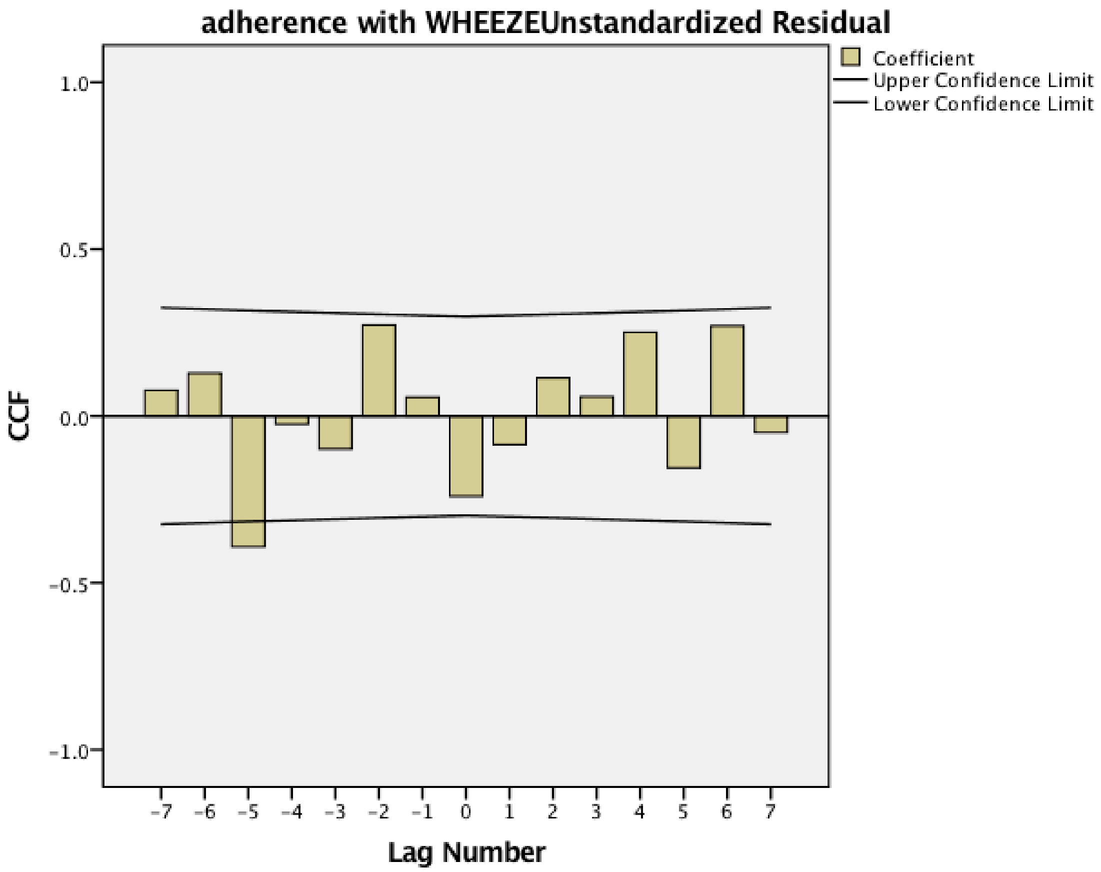
adherence with WHEEZEUnstandardized Residual
Coefficient
Upper Confidence Limit
Lower Confidence Limit
1.0
0.5
0.0
-0.5
-1.0
CCF
-7 -6 -5 -4 -3 -2 -1 0 1 2 3 4 5 6 7
Lag Number

Pain

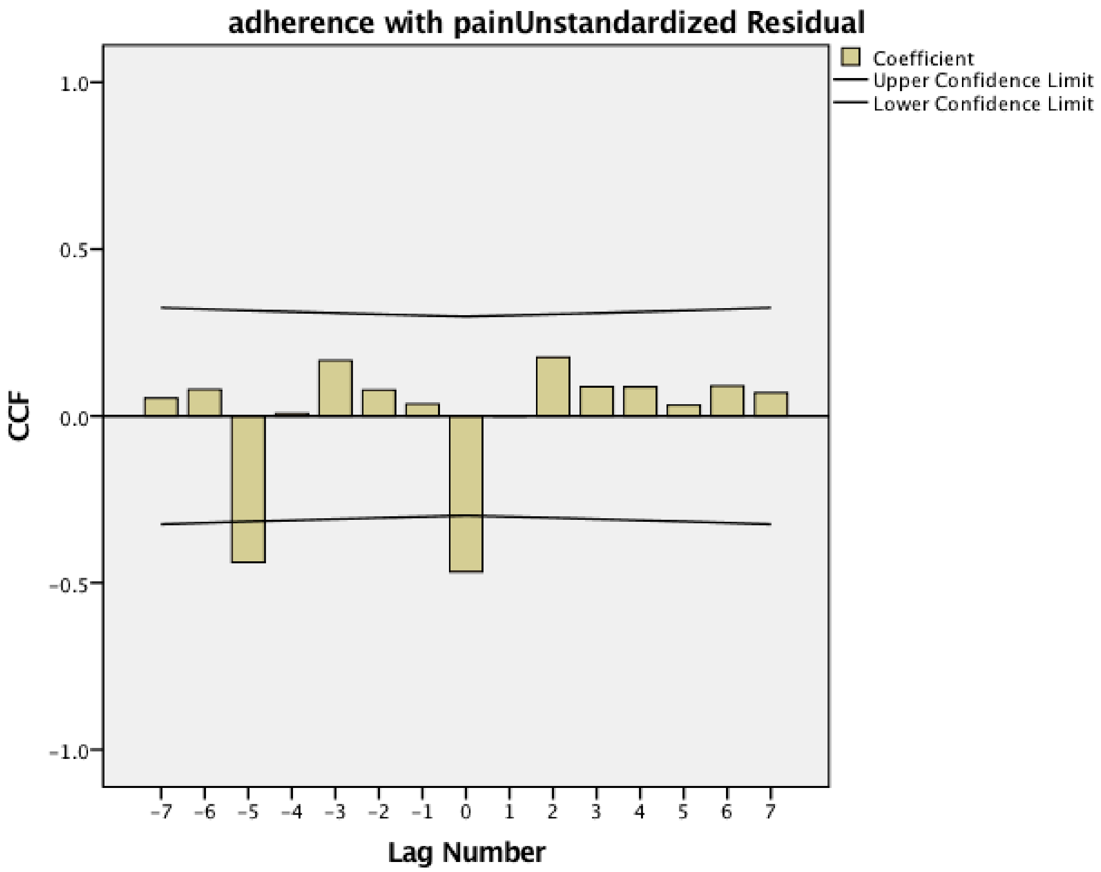
adherence with painUnstandardized Residual
Coefficient
Upper Confidence Limit
Lower Confidence Limit
CCF
1.0
0.5
0.0
-0.5
-1.0
-7
-6
-5
-4
-3
-2
-1
0
1
2
3
4
5
6
7
Lag Number

Tiredness

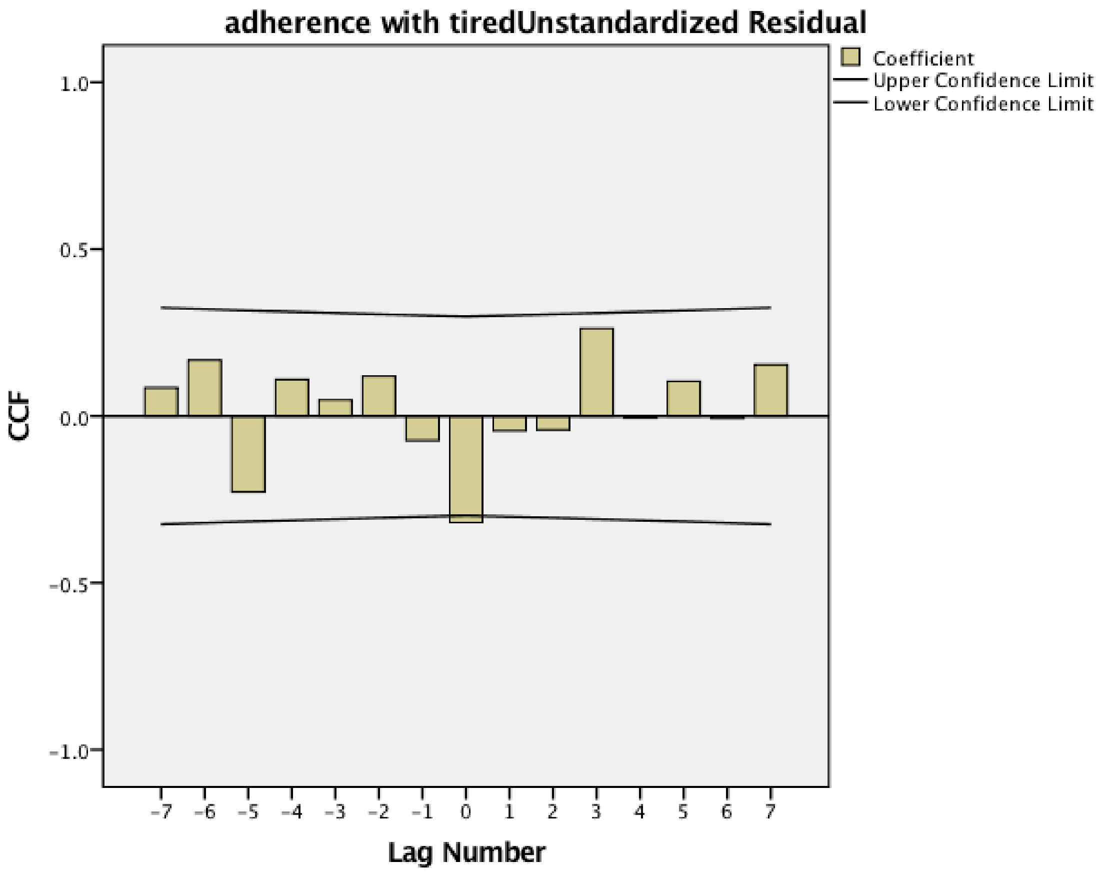

Participant 6

No cross correlations to report
